# Successful Nafamostat Mesilate Administration for Andexanet Alfa-Induced Heparin Resistance

**DOI:** 10.7759/cureus.44003

**Published:** 2023-08-23

**Authors:** Atsuhiro Kitaura, Tatsushige Iwamoto, Shinichi Hamasaki, Shota Tsukimoto, Yasufumi Nakajima

**Affiliations:** 1 Anesthesiology, Kindai University, Osaka, JPN; 2 Anesthesiology, Kanagawa Dental University, Yokosuka, JPN

**Keywords:** anticoagulation, anesthesia, direct oral anticoagulants, cardiopulmonary bypass, heparin resistance, andexanet alfa

## Abstract

Andexanet alfa is an analog of activated factor X and is used as an antagonist of anti-activated factor X agents. Andexanet alfa is useful for hemostasis in emergent bleeding during direct oral anticoagulant administration, which contributes to safety. In patients undergoing surgery with cardiopulmonary bypass because of heparin resistance, anesthesiologists are faced with a choice of anticoagulants. Herein, we experienced anesthesia for vascular prostheses with cardiopulmonary bypass for acute aortic dissection in a patient who had received andexanet alfa preoperatively. Heparin was initially used as the anticoagulant during cardiopulmonary bypass; however, despite the administration of large doses and antithrombin III preparations, anticoagulation was insufficient. Therefore, nafamostat mesilate was administered and sufficient anticoagulation was attained. The patient completed surgery under cardiopulmonary bypass, coagulation function was recovered shortly after withdrawal, and no obvious adverse effects were observed.

## Introduction

Andexanet alfa is an analog of activated factor X [1]. It is used as an antagonist of direct oral anticoagulants (DOACs). Andexanet alfa is useful for hemostasis in emergency bleeding during administration of DOACs and contributes to the safety of anticoagulation therapy with DOACs [1]. However, andexanet alfa is also known to neutralize the effects of heparin (heparin resistance) and should be administered with caution [2]. Inadequate anticoagulation during cardiopulmonary bypass (CPB) can lead to thromboembolism and circuit clotting, which can be fatal. Thus, andexanet alfa should not be used in patients scheduled for heparinized CPB [3]. However, anesthesiologists may respond to emergency surgery patients who are receiving andexanet alfa; thus, it is necessary to anticipate difficulties with anticoagulation during CPB. In this report, we describe our experience with anesthesia for aortic artificial vascular replacement under CPB for acute aortic dissection type A in a patient who had received andexanet alfa preoperatively, with discussion of the literature.

## Case presentation

**Figure 1:**
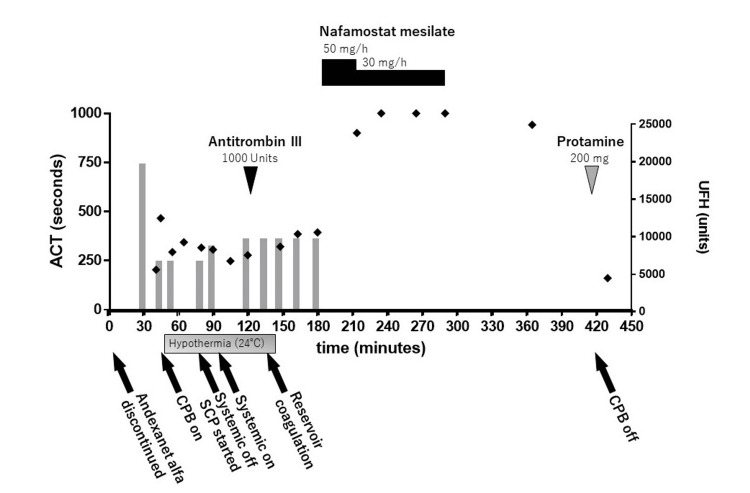
Medications and ACT around the CPB High-dose heparin administration and antithrombin supplementation do not adequately prolong ACT. Administration of nafamostat mesilate prolongs ACT sufficiently for CPB. Ninety-five minutes after the end of nafamostat mesilate administration, protamine is administered after withdrawal of CPB, and ACT is nearly normalized. ACT: activated clotting time, CPB: cardiopulmonary bypass, SCP: selective cerebral perfusion, UFH: unfractionated heparin

An 87-year-old woman (height 152 cm, weight 65 kg) with type A acute aortic dissection was admitted to our hospital. She was then brought into our operating room for an emergency total arch replacement. She had a history of deep vein thrombosis and was taking apixaban 10 mg/day. Andexanet alfa was started in the emergency room and administered continuously until it was discontinued when the patient entered the operating room. The preoperative blood test showed hemoglobin of 14.9 g/dl, platelets of 125,000/µL, and antithrombin III of 78%. Transthoracic echocardiography revealed a left ventricular ejection fraction of 0.64 with no significant valvular or pericardial effusion. General anesthesia was induced with 0.1 mg/kg midazolam and 200 μg fentanyl and maintained with 1-1.5% sevoflurane and 0.2-0.3 µg/kg/min remifentanil. Before CPB establishment, 300 units/kg unfractionated heparin (UFH) was administered. The activated clotting time (ACT) was prolonged to 466 seconds. Although the ACT was just short of 500 seconds, CPB was started with an additional 100 units/kg UFH at the surgeon's discretion. Thereafter, UFH was added as needed with frequent ACT checks; however, ACT remained around 250-350 seconds. Reservoir coagulation occurred 50 min after initiation of CPB. Seventy min after initiation of CPB, we administered 1000 units of antithrombin III, but ACT was prolonged to less than 400 seconds. Therefore, nafamostat mesilate 50 mg/h was started. Twenty-five minutes after initiation, ACT was prolonged to about 900 seconds. Nafamostat mesilate was reduced to 30 mg/h and continued. Sufficient prolongation of ACT was obtained thereafter. Aortic root and total arch replacement were performed combined with 25 minutes of systemic off and selective cerebral perfusion at 28 degrees Celsius. Thirty minutes prior to CPB withdrawal, nafamostat mesilate was discontinued. The duration of nafamostat mesilate administration was 90 minutes, and the total dose was 53.3 mg. After CPB withdrawal, protamine 3 mg/kg was administered, and ACT recovered to 161 seconds. The evolution of ACT around CPB and administration of anticoagulants are shown in Figure 1. However, soon after CPB withdrawal, surgical bleeding from the aorto-left coronary anastomosis occurred, and reanastomosis of the left coronary artery was performed with heart beating. During this process, wall motion abnormality in the anterior septal region appeared and left heart failure developed. We performed veno-arterial extracorporeal membrane oxygenation (VA-ECMO). The patient was transferred to the intensive care unit after surgery. Due to concerns about bleeding, anticoagulation for VA-ECMO was not performed. On postoperative day 2, her consciousness was confirmed. On postoperative day 5, an attempt was made to wean the patient from VA-ECMO but was abandoned due to poor oxygenation by the autologous lungs. Seven days postoperatively, the patient passed away due to accidental removal of the arterial cannula of VA-ECMO.

## Discussion

We experienced a case of heparin resistance in a patient receiving andexanet alfa. The patient was able to continue CPB with nafamostat mesilate.

Andexanet alfa is an antagonist of factor Xa agents [4], and it rapidly neutralizes anti-Xa factor inhibitors [1,5] such as DOACs and UFH. Andexanet alfa can act as an antagonist to DOACs during emergent bleeding [5], which contributes to the safety of anticoagulation therapy with DOACs. Since andexanet alfa is a decoy for factor Xa, it also binds to antithrombin. The antithrombin-andexanet alfa complex binds to UFH but does not activate anticoagulant effects. Heparin resistance is presumed to occur due to depletion of UFH and antithrombin. UFH is commonly used as an anticoagulant in CPB. Decreased efficacy of UFH may result in coagulation of the CPB circuit and thromboembolism, which can be fatal. Therefore, the use of andexanet alfa in patients scheduled for surgery with heparin is not recommended [3]. In the present case, the use of andexanet alfa by a cardiologist to stop bleeding preoperatively was not appropriate.

When emergency surgery is required after andexanet alfa, anesthesiologists need to consider the possibility of heparin resistance due to andexanet alfa. There is a report that the use of 80,000 units of heparin prolonged ACT and allowed CPB surgery without complications in a patient receiving andexanet alfa [6]. In addition, it has been reported that 1000 units of antithrombin III prolonged ACT in a patient after the administration of andexanet alfa [7]. UFH and antithrombin supplementation ameliorate the UFH and antithrombin deficiency caused by andexanet alfa. As a result, the formation of the factor Xa-antithrombin-antithrombin-heparin complex was promoted, possibly prolonging ACT [7]. These reports suggest that wastage of heparin and antithrombin is the mechanism of andexanet alfa-induced heparin resistance. However, in the present case, 27,000 units of heparin were used at the start of CPB followed by 120,000 units during an approximately two-hour period, without sufficient prolongation of ACT. Antithrombin supplementation was also insufficient to prolong ACT. In the present case, consumption coagulopathy may have progressed rapidly due to acute aortic dissection, resulting in insufficient antithrombin supplementation. Adequate prolongation of ACT and anticoagulation with UFH were not achieved; therefore, the use of anticoagulants other than UFH was considered. Argatroban is a direct-activated factor Xa inhibitor used in heparin-induced thrombocytopenia that exerts its anticoagulant function without antithrombin [8]. In the present case, we did not administer argatroban, as it has a long half-life of about 30 minutes and no antagonist, so there is a risk of uncontrolled hemorrhage in major vascular surgery [9]. Instead, we administered nafamostat mesilate, an anticoagulant used in dialysis. Its molecular weight is about 540 Da [10]. Nafamostat mesilate has a short half-life of eight minutes and is likely to improve coagulation quickly after administration [10]. Nafamostat mesilate directly inhibits proteolytic enzymes such as VIIa, Xa, thrombin, kallikrein, platelet aggregation, plasmin, complement, and trypsin. Antithrombin is not involved in the anticoagulant effect of nafamostat mesilate [10]. Nafamostat mesilate exerts its anticoagulant effect by acting on many points of action regardless of antithrombin. Therefore, it was considered to have sufficient anticoagulant activity even in the presence of andexanet. Using nafamostat mesilate as an anticoagulant in CPB in combination with small doses of heparin [11] or aprotinin [12] has already been reported, and it is expected to maintain platelet function during CPB and reduce perioperative bleeding complications. In addition, there is already one report on the safe use of nafamostat mesilate in patients with heparin resistance [13]. However, the study did not discuss the cause of heparin resistance. Our case demonstrates the possibility of using nafamostat mesilate as an anticoagulant when CPB must be performed in patients who develop heparin resistance with andexanet alfa.

## Conclusions

We experienced a patient with heparin resistance due to andexanet alfa who did not improve with additional heparin or antithrombin but was able to maintain CPB with nafamostat mesilate as an alternative anticoagulant to heparin. Nafamostat mesilate may be useful as an anticoagulant during CPB in patients who develop heparin resistance with andexanet alfa. 
